# Feeding and Eating Disorder and Risk of Subsequent Neurodevelopmental Disorders: A Population-Based Cohort Study

**DOI:** 10.3389/fped.2021.671631

**Published:** 2021-09-06

**Authors:** Hongyun Shan, Fei Li, Jun Zhang, Hui Wang, Jiong Li

**Affiliations:** ^1^Ministry of Education -Shanghai Key Laboratory of Children's Environmental Health, Xin Hua Hospital Affiliated to Shanghai Jiao Tong University School of Medicine, Shanghai, China; ^2^Department of Clinical Medicine, Aarhus University, Aarhus, Denmark; ^3^Department of Clinical Epidemiology, Aarhus University, Aarhus, Denmark; ^4^Department of Developmental and Behavioral Pediatric & Child Primary Care, Xin Hua Hospital Affiliated to Shanghai Jiao Tong University School of Medicine, Shanghai, China

**Keywords:** feeding and eating disorder, neurodevelopmental disorders, register-based research, cohort study, attention-deficit/hyperactivity disorder, autism spectrum disorder, intellectual disability

## Abstract

**Background:** There are limited data concerning the long-term mental health of children with feeding and eating disorder (FED). We aimed to investigate whether children with FED are at greater risks of developing emotional/behavioral disorders with onset usually during childhood, attention-deficit/hyperactivity disorder (ADHD), autism spectrum disorder (ASD), and intellectual disability (ID).

**Methods:** We conducted a population-based cohort study, including all singleton births in Denmark from January 1, 1995, to December 31, 2015. For each child diagnosed with FED, 10 age- and sex-matched controls who did not meet the criteria for FED were randomly selected from the general population. Associations were estimated with Cox regression modes adjusting for other perinatal and maternal factors, and sibling analyses were performed for controlling potential confounding by shared familial (genetic or environmental) factors.

**Results:** Of the 1,256,989 individuals in the cohort, there were 1967 (53.4% girls) children diagnosed with FED. Children with FED had higher risks for clinically diagnosed emotional/behavioral disorders with onset usually in childhood (hazard ratio [HR], 2.78; 95% CI, 2.34–3.31), ADHD (HR, 1.74; 95% CI, 1.33–2.26), ASD (HR, 3.05; 95% CI, 2.36–3.94), and ID (HR, 6.38; 95% CI, 4.48–9.11), compared with matched controls. Girls with FED are at greater risks for emotional/behavioral disorders and ID, but not ADHD and ASD. Alike, in sibling analysis, increased rates are also observed for other neurodevelopmental disorders, but not for ADHD.

**Conclusion:** Children with FED are associated with substantially increased risks of emotional/behavioral disorders, ADHD, ASD, and ID. This study highlights the importance of carefully monitoring neurodevelopmental disorders in children with FED.

## Introduction

Feeding and eating disorder (FED) is a neurodevelopmental disorder, typically manifested in the first years of life (1). FED affects ~0.8–1.4% of the normally developing children and is more common in girls (2). FED is characterized by persistent eating disturbances in the presence of an adequate food supply, and eating-related problems like voluntary regurgitation (3). The development of FED is thought to be affected by interactions of both genetic components and early environmental factors (1, 4).

Previous studies have shown that children with FED are at greater risks for other neurodevelopmental symptoms, including cognitive deficits, developmental delays, behavioral inhibition, and social difficulties (5–7). More importantly, FED usually occurs during early infancy while other neurodevelopmental disorders, such as attentional-deficit/hyperactivity disorders (ADHD), emerge in childhood. Several prospective longitudinal studies found that feeding disorders were associated with increased risks of inattention and emotional and internalizing and externalizing behavioral problems during childhood (8–11). However, these previous studies are limited by relatively small sample size and focusing on psychiatric symptoms using rating scales rather than clinical diagnosed psychiatric disorders (6–10). It is still unknown whether FED is associated with increased risks of neurodevelopmental disorders usually diagnosed during childhood in clinical settings or hospitals. In addition, previous studies often failed to account for the role of unmeasured familial factors, such as shared environmental or genetic factors (12).

Neurodevelopmental disorders have been associated with low functioning in academic, socially disadvantaged environments (13). Understanding the risk of comorbid emotional and behavioral problems is important to monitor the long-term outcomes for children diagnosed with FED. We therefore conducted a large, longitudinal, population-based cohort study to explore the risks of clinical diagnosed emotional/behavioral disorders, ADHD, autism spectrum disorder (ASD), and intellectual disability (ID) in children with FED compared with age- and sex-matched controls. We further examined whether these associations varied by sex and used a sibling comparison analysis that controlled for possible shared familial confounders.

## Methods

### Data Source and Cohort Identification

**Figure 1:**
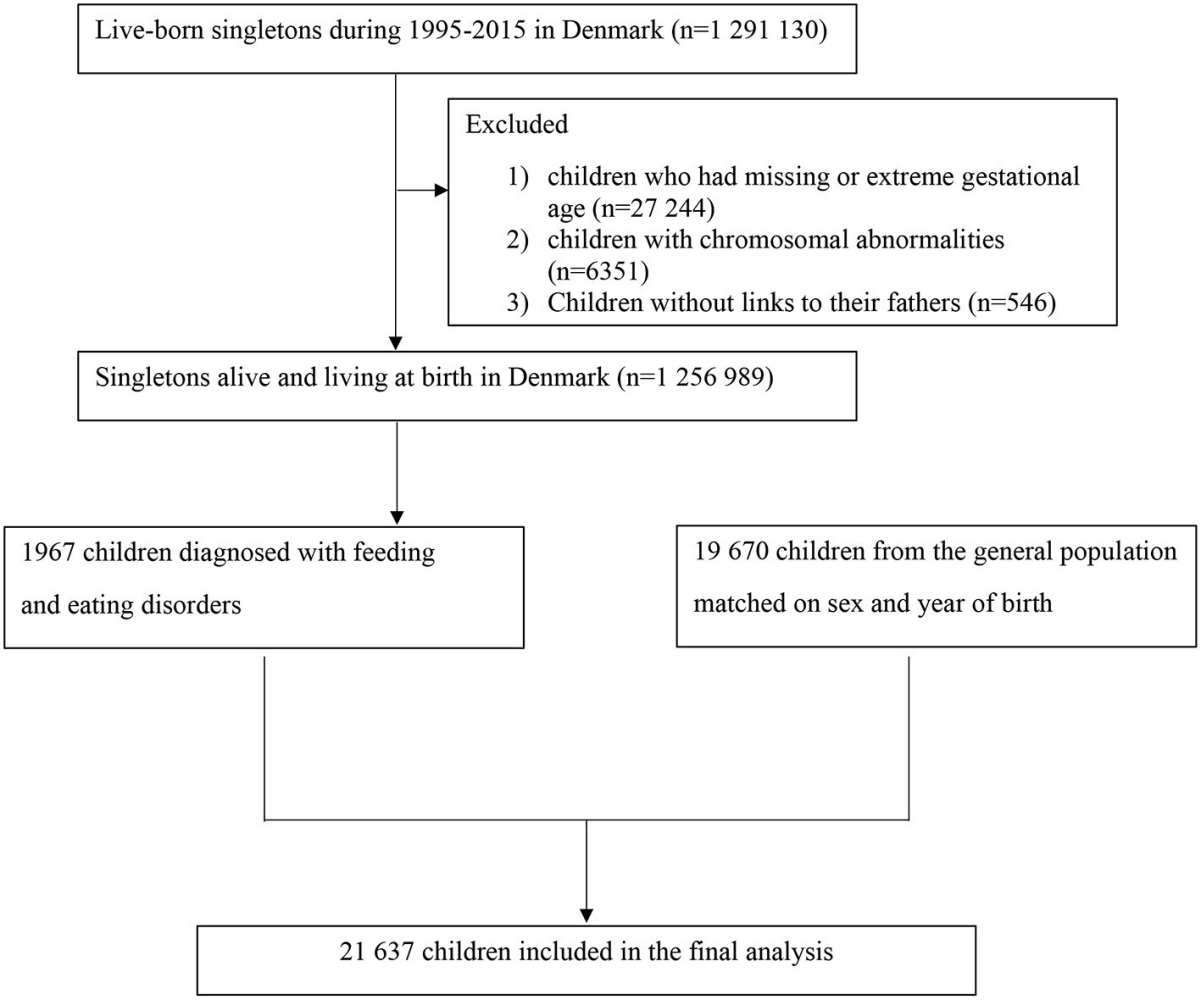
Overview of the study population.

We identified all singleton live births in Denmark from January 1, 1995, to December 31, 2015 (*n* = 1,291,130) via linkage between the Danish Civil Registration Service and Medical Birth Register. All live births and residents are assigned a unique personal national identification number, which can be used for accurate linkage of other nationwide administrative registers (14–18). We excluded 27,244 children who had invalid gestational age (<154 days or >315 days), 6,351 children diagnosed with chromosomal abnormalities, and 546 children without linkage to their fathers (shown in Figure 1). We followed each child from birth until the outcome of interest, emigration, death, or end of follow up (December 31, 2016), whichever came first. The study was approved by the Danish Data Protection Agency (No 2013-41-2569). The requirement for individual informed consent was not applied for a register-based study in Denmark.

### Feeding and Eating Disorder (FED)

Information on FED was obtained from the Danish National Patient Register (DNPR) and the Danish Psychiatric Central Research Register (15, 16), which hold the diagnostic information on all inpatient and outpatient psychiatric disorders in Denmark. In Denmark, the *International Statistical Classification of Disease and Related Health problems, 10th revision* (*ICD-10*) was used from 1995 onwards. The *ICD-10* codes for FED were F98.2 and F50.8 (1, 19). The cut-off age for receiving a diagnosis of FED was 3 years (1). For each of the children diagnosed with FED, we randomly selected 10 control subjects, who were matched individually with respect to birth year and sex. The matched controls did not have a FED diagnosis in the DNRP or the Danish Psychiatric Central Research Register and were still alive in Denmark when the children with FED received the diagnosis.

### Ascertainment of Outcomes

Information on neurodevelopmental disorders was obtained from the DNRP and the Danish Psychiatric Central Research Register (15, 16). The outcomes of interest included (1) emotional/behavioral disorders with onset usually in childhood (*ICD-10*: F90-F98 excluding F98.2); (2) ADHD (*ICD-10*: F90.0 and F98.8); (3) ASD (*ICD-10*: F84.0, F84.1, F84.5, F84.8 and F84.9); (4) ID (*ICD-10*: F70-F79) (20, 21). When investigating the specific neurodevelopmental disorders, we defined the date of onset as the first day of each specific diagnosis, irrespective of other neurodevelopmental disorders diagnoses, if they existed. The full denomination of these diagnoses was indicated in Supplementary Table 1.

### Covariates

Based on previous research (1, 22–24), the following covariates were considered as potential confounders and included in the adjusted model: parity (1, 2, ≥3), maternal age at birth (≤25, 26–30, 31–35, ≥36 years), paternal age at birth (≤25, 26–30, 31–35, ≥36 years), maternal country of origin (Denmark, other countries), maternal education level (0–9, 10–14, ≥15 years), maternal cohabitation status (yes, no), maternal smoking status during early pregnancy (yes, no), maternal psychiatric disorder history before childbirth (yes, no) (*ICD-8*: 290-315; *ICD-10*: F00-F98), and paternal psychiatric disorder history (yes, no) (*ICD-8*: 290-315; *ICD-10*: F00-F98).

### Statistical Analysis

We used Cox proportional hazards regression analyses to estimate hazard ratios (HRs) and corresponding 95% confidence intervals (CIs) for emotional/behavioral disorders, ADHD, ASD, and ID in exposed children (those with a diagnosis of FED) compared with unexposed children (those without a diagnosis of FED), with age as the underlying time scale. A robust sandwich estimator function was used to account for clustering of individuals within nuclear families (i.e., sibling bound by the same biological mother). The test for comparing the covariates was χ^2^ test for proportions. We also investigated whether gender modified the associations by including the interaction term between FED and gender using the Wald test.

To account for unmeasured familial confounding, sibling analyses were conducted using stratified Cox regression with a separate stratum for each family as identified by their mother's anonymous identification number (25). In the stratified Cox model, each family had its own baseline hazard rate, reflecting the effects of the shared genetic and environmental characteristics. We then compared the children with FED with their sibling(s) with regard to the subsequent neurodevelopmental disorders, and further adjusting for all of the covariates as in main analyses.

We performed several sensitivity analyses: (1) to examine whether the associations were modified by maternal psychiatric disorders, we conducted the analyses stratified by maternal psychiatric disorder before the childbirth (yes, no); (2) to ascertain the potential confounding effect by preterm birth, we performed the analyses stratified by preterm birth; (3) to exclude the confounding effect of neonatal complications, we conducted the analyses by excluding children born with low birth weight or low Apgar score at 5 min (<7); (4) we repeated our analyses by excluding children diagnosed with cleft lip and cleft palate (*ICD-10*: Q35-37) and other congenital malformations of the digestive system (*ICD-10*: Q38-45); (5) we performed analyses by using the general population cohort (*n* = 1,256,989). All statistical analyses were performed using STATA, version 15.1 (StataCorp).

## Results

We identified a total of 1,967 children diagnosed with FED (917 boys and 1,050 girls). The prevalence of FED in this study was 0.6%. The median age at first diagnosis of FED was 0.6 years (interquartile range, 0.3–1.2 years). The median follow-up is 6.3 years (interquartile range, 3.4–9.3 years). Baseline characteristics of children with FED and their matched control subjects (*n* = 19,670) are presented in Table 1. Children with FED were more often to be preterm and firstborn. Parents of children with FED were more likely to be younger at the time of childbirth. Mothers of children with FED were less likely to be of Danish origin and had lower level of education and more comorbid psychiatric disorders.

**Table 1:** Baseline characteristics for children with feeding and eating disorder and matched controls.

**Characteristics**	**Matched control**	**Feeding and eating disorder**	***p***
	***n* (%)**	***n* (%)**	
**Sex**			
Boys	9,170 (46.6)	917 (46.6)	
Girls	10,500 (53.4)	1,050 (53.4)	
**Year of birth**			
1995–1997	2,550 (13.0)	255 (13.0)	
1998–2000	1,720 (8.7)	172 (8.7)	
2001–2003	2,710 (13.8)	271 (13.8)	
2004–2006	2,910 (14.8)	291 (14.8)	
2007–2009	3,400 (17.3)	340 (17.3)	
2010–2012	3,680 (18.7)	368 (18.7)	
2013–2015	2,700 (13.7)	270 (13.7)	
**Preterm birth (<37 gestational weeks)**			
No	18,687 (95.0)	1,709 (86.9)	<0.001
Yes	983 (5.0)	258 (13.1)	
**Parity**			
1	8,714 (44.3)	1,063 (54.0)	<0.001
2	7,340 (37.3)	604 (30.7)	
≥3	3,616 (18.4)	300 (15.3)	
**Paternal age (years)**			
≤24	1,856 (9.5)	221 (11.4)	0.001
25–29	5,509 (28.3)	605 (31.1)	
30–34	6,790 (34.9)	619 (31.9)	
≥35	5,307 (27.3)	498 (25.6)	
**Maternal age (years)**			
≤24	2,644 (13.4)	332 (16.9)	<0.001
25–29	6,561 (33.4)	688 (35.0)	
30–34	6,930 (35.2)	639 (32.5)	
≥35	3,535 (18.0)	308 (15.6)	
**Maternal education level (years)**			
≤9	3,555 (18.5)	493 (25.9)	<0.001
10–15	8,698 (45.3)	828 (43.4)	
>15	6,935 (36.2)	585 (30.7)	
**Maternal cohabitation status at birth**			
No	9,410 (47.9)	923 (47.0)	0.444
Yes	10,245 (52.1)	1,042 (53.0)	
**Maternal origin**			
Not born in Denmark	2,837 (14.4)	473 (24.0)	<0.001
Born in Denmark	16,826 (85.6)	1,494 (76.0)	
**Maternal psychiatric disorders**			
No	17,938 (91.2)	1,643 (83.5)	<0.001
Yes	1,732 (8.8)	324 (16.5)	
**Paternal psychiatric disorders**			
No	18,317 (93.1)	1,806 (91.8)	0.03
Yes	1,353 (6.9)	161 (8.2)	
**Maternal smoking status during pregnancy**			
No	15,902 (83.0)	1,533 (80.4)	0.004
Yes	3,264 (17.0)	375 (19.6)	

Children with FED had substantially increased risks for receiving diagnoses for emotional/behavioral disorders with onset usually in childhood (HR 2.78, 95% CI [2.34–3.31]), ADHD (HR 1.74, 95% CI [1.33–2.26]), ASD (HR 3.05, 95% CI [2.36–3.94]), and ID (HR 6.38, 95% CI [4.48–9.11]). The increased risk for emotional/behavioral disorders in individuals with FED was statistical significantly higher among girls than boys (HR 4.05, 95% CI [3.12–5.25] vs. HR 2.16, 95% CI [1.70–2.73], *p* value for interaction: <0.001), and also for ID (HR 9.73, 95% CI [5.48–17.26] vs. HR 4.94, 95% CI [3.10–7.86], *p* value for interaction: <0.001). The increased risks of ADHD (HR 2.01, 95% CI [1.29–3.15] vs. HR 1.62, 95% CI [1.17–2.25]) and ASD (HR 4.54, 95% CI [2.90–7.12] vs. HR 2.58, 95% CI [1.88–3.53]) in individuals with FED were also higher among girls compared with boys, although the difference was not statistically significant (*p* values for interaction were 0.17 and 0.18, respectively) (shown in Table 2).

**Table 2:** Risk of neurodevelopmental disorders in individuals diagnosed with feeding and eating disorder compared with age and sex matched controls (*n* = 21,637).

	**Individuals with FED**		**Matched controls**		**Unadjusted** **HR (95% CI)**	**Adjusted^#^** **HR (95% CI)**
	**No. diagnosed**	Incidence rate^*^	**No. diagnosed**	Incidence rate^*^		
**All combined**						
Emotional/behavioral disorder	175	8.92	612	3.03	2.98 (2.52–3.52)	2.78 (2.34–3.31)
Attention-deficit/hyperactivity disorder	68	3.33	369	1.82	1.85 (1.43–2.40)	1.74 (1.33–2.26)
Autism spectrum disorder	84	4.12	261	1.28	3.24 (2.53–4.14)	3.05 (2.36–3.94)
Intellectual disability	60	2.89	77	0.38	7.67 (5.47–10.75)	6.38 (4.48–9.11)
**Boys**						
Emotional/behavioral disorder	90	9.80	405	4.26	2.33 (1.86–2.93)	2.16 (1.70–2.73)
Attention-deficit/hyperactivity disorder	43	4.49	254	2.64	1.72 (1.24–2.37)	1.62 (1.17–2.25)
Autism spectrum disorder	54	5.63	197	2.04	2.77 (2.05–3.74)	2.58 (1.88–3.53)
Intellectual disability	32	3.25	53	0.54	5.74 (3.70–8.92)	4.94 (3.10–7.86)
**Girls**						
Emotional/behavioral disorder	85	8.14	207	1.94	4.26 (3.31–5.48)	4.05 (3.12–5.25)
Attention-deficit/hyperactivity disorder	25	2.31	115	1.07	2.18 (1.41–3.36)	2.01 (1.29–3.15)
Autism spectrum disorder	30	2.78	64	0.60	4.70 (3.04–7.24)	4.54 (2.90–7.12)
Intellectual disability	28	2.56	24	0.22	12.12 (7.02–20.92)	9.73 (5.48–17.26)

*FED, feeding and eating disorders; No., number; HR, hazard ratio; CI, confidence interval*;

* *incident rate per 1,000 person-years*;

# *adjusted for parity, parental age, maternal education, maternal origin, maternal cohabitation at birth, maternal smoking status during early pregnancy, parental history of psychiatric disorders before childbirth, and pregnancy complications including diabetes and pre-eclampsia*.

Of the 408,397 families with at least 2 singleton siblings, 1,370 families included siblings who were discordant for FED. In the sibling comparison analyses, the risk estimate for ADHD was reduced but not eliminated (HR 1.17, 95% CI [0.78–1.77]). Compared to children without FED, children with FED had higher risks for other neurodevelopmental disorders, for example, the HRs for emotional/behavioral disorders, ADHD, ASD, and ID were 1.63 (95% CI [1.24–2.13]), 1.17 (95% CI [0.78–1.77]), 2.20 (95% CI [1.43–3.38]), and 3.76 (95% CI [2.20–6.42]), respectively (shown in Table 3).

**Table 3:** Sibling comparison of neurodevelopmental disorders in individuals diagnosed with feeding and eating disorder (*n* = 3,347).

	**Individuals with FED**		**Siblings without FED**		**Adjusted for birth year and sex** **HR (95% CI)**	**Adjusted^#^** **HR (95% CI)**
	**No. diagnosed**	Incidence rate^*^	**No. diagnosed**	Incidence rate^*^		
**Neurodevelopmental disorders**						
Behavioral/emotional disorders	113	8.29	109	5.44	1.55 (1.19–2.02)	1.63 (1.24–2.13)
Attention-deficit/hyperactivity disorder	41	2.90	57	2.80	1.13 (0.76–1.69)	1.17 (0.78–1.77)
Autism spectrum disorder	53	3.75	36	1.76	2.22 (1.45–3.36)	2.20 (1.43–3.38)
Intellectual disability	43	3.06	19	0.93	3.29 (1.96–5.50)	3.76 (2.20–6.42)

*FED, feeding and eating disorders; No., number; HR, hazard ratio; CI, confidence interval*;

* *incident rate per 1,000 person-years*;

# *adjusted for parity, parental age, maternal education, maternal origin, maternal cohabitation at birth, maternal smoking status during early pregnancy, parental history of psychiatric disorders before childbirth, and pregnancy complications including diabetes and pre-eclampsia*.

Stratification analyses by maternal psychiatric disorders and preterm birth showed similar results (Supplementary Tables 2, 3). The analyses excluding children born with neonatal outcomes or with cleft lip and cleft palate and other congenital malformations of the digestive system, or using the general population cohort, were similar to the main findings (Supplementary Tables 4–6).

## Discussion

To our knowledge, this is the first nationwide, prospective study to report the risk of clinically diagnosed neurodevelopmental disorders among children with FED. We observed substantially increased risks of emotional/behavioral disorders, ADHD, ASD, and ID in children with FED compared to the matched controls, after adjustment for potential confounders. The associations were stronger in girls than in boys and remained in sibling comparison analyses.

The findings are novel because previous studies have investigated feeding behavioral problems rather than clinical diagnoses of FED. Our findings are in line with previous studies using questionnaires, indicating that children with FED have increased risks of other neurodevelopmental disorders in later life (6–9, 11). The psychosocial impairments associated with children with FED, including developmental delays, adaptive communication, socialization, and irritable/disinhibited behavioral, are problematic to manage and particularly increase the risks of the emotional and behavioral disorders (4, 9, 26–28). FED is also associated with cognitive deficits, which in turn increased the risk of ID (29, 30). Additionally, FED has associated with physical issues, such as neuromuscular disorders, gastrointestinal disorders, and food allergies, which contribute to the development of neurodevelopmental disorders (31, 32). This study expands our understanding by indicating that children with FED have an increased risk of a wide range of neurodevelopmental disorders.

Sex appears to play an important role in the association between FED and neurodevelopmental disorders. Specifically, girls with FED have greater risks for these neurodevelopmental disorders, compared to boys with FED. Previous research has demonstrated that females diagnosed with one mental disorder may be associated with greater risks of other psychiatric illnesses (33, 34). Being female with ASD may be associated with a greater risk for emotional disorder compared with their male counterparts (35). Similarly, another study has shown that girls with depressive disorder were at greater risks for anxiety or suicidal attempts (36).

The non-specific pattern, i.e., all these examined neurodevelopmental disorders are associated with FED, is congruent with genome-wide association studies reporting an overlap of genetic factors across the neurodevelopmental disorders, including ASD and ID (37). Potential shared genetic liability among FED, ADHD, ASD, and other emotional/behavioral disorders may also contribute to the observed associations (38). Moreover, it has also been proposed that children with ADHD, ASD, or other emotional/behavioral disorders have shared neurocognitive endophenotypes, such as visual spatial short-term working memory and deficits in executive function (39).

Sibling analyses allowed us to further control for shared environmental and genetic confounders (40). The estimates from sibling comparison analyses were attenuated but are generally consistent with the findings from the population-based study. For ADHD, even though the confidence intervals were wide due to the small number of discordant siblings, the risk was still 17% higher for individuals with FED. The reduced estimates indicate that the associations could also be partly attributed to unmeasured confounding shared among siblings.

We observed little evidence that the association is explained by increased risk for neonatal complications among children with FED. Of note, children diagnosed with FED were more likely to be born preterm (1). Preterm birth has been shown to be a risk factor for neurodevelopmental disorders (41). However, this finding was also seen when restricting the analysis to term births, suggesting that the association was not explained by preterm birth. Further, after excluding children born with low birth weight and low Apgar score at 5 min, the results remained similar, suggesting these associations were not driven by adverse neonatal characteristics.

Our study had a number of strengths. First, this study was a population-based study using national registers with nearly complete follow-up. Thus, the data are representative of the whole population and the findings can be generalized to similar populations. Second, all information was actually obtained in a prospective and objective manner, which precluded recall bias (14, 17). Third, the registers allowed for adjustment for a number of potentially important confounders, such as parental age, parental history of psychiatric disorders, maternal education level, maternal country of birth, sex, parity, and calendar year of birth. Fourth, because control subjects were selected randomly from a population-based birth cohort, selection bias for control subjects is highly unlikely. Fifth, we used a sibling control design to further minimize the influence of unmeasured confounding and similar results were found between the population and sibling comparison analysis.

Several limitations should be noted. First, the prevalence of FED was low compared to other population-based studies in other countries like the United States (4, 11). However, when compared to other studies using the hospital-based diagnoses extracted from the National Registers (1, 2), the prevalence was similar. Although register-based diagnoses are objective and not subject to recall bias, we might have only captured children with severe FED because children with mild FED might not seek help, which could lead to misclassification of exposure. Nevertheless, the misclassification of FED is non-differential and most likely will bias the results toward the null, because FED diagnosis was made before outcome ascertainment. Second, it is reasonable to assume that children with FED means more regular contact with health-care services, which in turn may affect the recognition and the registration of other neurodevelopmental disorders more often than children without FED, causing an overestimation due to detection bias. Nevertheless, these children were followed from birth through the childhood and all children with neurodevelopmental disorders will be diagnosed. Therefore, detection bias is unlikely to explain the observed association. Third, even though sibling analyses were conducted to control for shared genetic and/or environmental factors among sibling, we could not rule out the possibility that the association can be entirely explained by genetic confounding.

Our findings have public health importance, suggesting that children diagnosed with FED may benefit from anticipatory screening and treatment for neurodevelopmental disorders. Neurodevelopmental disorders, especially ASD and ID, are a group of conditions with onset during the developmental period, which are associated with impairments in personal, social, and academic functioning (13). Caring for children with neurodevelopmental disorders can also be disruptive to family life and often causes considerable stress for parents and others who live with them. Early intervention is essential to improve the developmental trajectory. Our study suggests that FED during infancy can be considered as a reliable sign for early identification. From a public health perspective, early identification of these children to ensure that they could receive timely services and special education program is of critical importance.

## Conclusion

Our findings suggest that children with FED are at substantially increased risks of clinical diagnosed neurodevelopmental disorders during childhood. These results support recommendations on early neurodevelopmental disorders surveillance in children with FED, especially in girls.

## Data Availability Statement

The original contributions presented in the study are included in the article/Supplementary Materials, further inquiries can be directed to the corresponding authors.

## Ethics Statement

The studies involving human participants were reviewed and approved by the study was approved by the Danish Data Protection Agency (No 2013-41-2569). Written informed consent to participate in this study was not required in accordance with the local legislation and institutional requirements.

## Author Contributions

HS performed the literature review, conducted data analyses, and drafted the manuscript under the supervision of HW. JL had full access to all of the data in this study and takes responsibility for the integrity of the data and the accuracy of the data analysis. JZ contributed to the interpretation of the data and critical revision of the paper for important intellectual content. FL helped in the analyzing the data and gave suggestions on the current manuscript. All authors approved the final manuscript as submitted and agreed to be accountable for all aspects of the work.

## Conflict of Interest

The authors declare that the research was conducted in the absence of any commercial or financial relationships that could be construed as a potential conflict of interest.

## Publisher's Note

All claims expressed in this article are solely those of the authors and do not necessarily represent those of their affiliated organizations, or those of the publisher, the editors and the reviewers. Any product that may be evaluated in this article, or claim that may be made by its manufacturer, is not guaranteed or endorsed by the publisher.

## Abbreviations

FED: feeding and eating disorder
ADHD: attention-deficit/hyperactivity disorder
ASD: autism spectrum disorder
ID: intellectual disability
HR: hazard ratio
CI: confidence intervals
ICD-10: International Statistical Classification of Disease and Related Health problems, 10th revision
DNPR: Danish National Patient Register.
