# Supplementary material for: Feeding and Eating Disorder and Risk of Subsequent Neurodevelopmental Disorders: A Population-Based Cohort Study
Source: Front Pediatr. 2021 Sep 6;9:671631. doi: 10.3389/fped.2021.671631 (PMC8451269; doi:10.3389/fped.2021.671631)
Supplement: Supplementary file 1 [file Data_Sheet_1.docx]

Supplementary Table 1 The full denomination of diagnosis for the neurodevelopmental disorders

| Diagnosis | *ICD*-10 code | Denomination |
| --- | --- | --- |
| Emotional/behavioral disorders | F90-98 excluding F98.2 | (1) Hyperkinetic disorders (Disturbance of activity and attention, hyperkinetic conduct disorder, other hyperkinetic disorders, hyperkinetic disorder, unspecified)  (2) Conduct disorders (conduct disorders confined to the family context, unsocialized conduct disorder, socialized conduct disorder, oppositional defiant disorder, other conduct disorders, conduct disorder, unspecified)  (3) Mixed disorders of conduct and emotions (Depressive conduct disorder, other mixed disorders of conduct and emotions, mixed disorder of conduct and emotions, unspecified)  (4) Emotional disorders with onset specific to childhood (separation anxiety disorder of childhood, phobic anxiety disorder of childhood, social anxiety disorder of childhood, sibling revalry disorder, other childhood emotional disorders, childhood emotional disorder, unspecified  (5) Disorders of social functioning with onset specific to childhood and adolescence (elective mutism, reactive attachment disorder of childhood, disinhibited attachment disorder of childhood, other childhood disorder of social functioning, childhood disorder of social functioning, unspecified)  (6) Tic disorders (Transient tic disorders, chronic moto or vocal tic disorder, combined vocal and multiple motor tic disorder, other tic disorders, tic disorder, unspecified)  (7) Other behavioural and emotional disorders with onset usually occurring in childhood and adolescence (nonorganic enuresis, nonorganic encopresis, pica of infancy and childhood, stereotyped movement disorders, stuttering, cluttering, other specified behavioural and emotional disorders with onset usually occurring in childhood and adolescence, unspecified behavioural and emotional disorders with onset usually occurring in childhood and adolescence. |
| Attention-deficit/hyperactivity disorder | F90.0 and F98.8 | (1) Attention deficit with disorder with hyperactivity, hyperactivity disorders, syndrome with hyperactivity;  (2) Attention deficit disorder without hyperactivity |
| Autism spectrum disorder | F84.0, F84.1, F84.5, F84.8 and F84.9 | (1) The presence of abnormal or impaired development that is manifest before the age of three years, and the characteristic type of abnormal functioning in all the three areas of psychopathology: reciprocal social interaction, communication, and restricted, stereotyped, repetitive behavior. In addition to these specific diagnostic features, a range of other nonspecific problems are common, such as phobias, sleeping and eating disturbances, temper tantrums, and (self-directed) aggression.  (2) A type of pervasive developmental disorder that differs from childhood autism either in age of onset or in failing to fulfill all three sets of diagnostic criteria. The subcategory should be used when there is abnormal and impaired development that is present only after age three years, and a lack of sufficient demonstrable abnormalities in one or two of the three areas of psychopathology required for the diagnosis of autism (namely, reciprocal social interactions, communication, and restricted, stereotype, repetitive behaviour) in spite of characteristic abnormalities in the other area(s). Atypical autism arises most often in profoundly retarded individuals and in individuals with a severe specific developmental disorder of receptive language.  (3) A disorder of uncertain nosologically validity, characterized by the same type of qualitative abnormalities of reciprocal social interaction that typify autism, together with a restricted, stereotyped, repetitive repertoire of interests and activities. It differs from autism primarily in the fact that there is no general delay or retardation in language or in cognitive development. This disorder is often associated with marked clumsiness. There is a strong tendency for the abnormalities to persist into adolescence and adult life. Psychotic episodes occasionally occur in early adult life.  (4) other pervasive developmental disorders  (5) Pervasive developmental disorder, unspecified |
| Intellectual disability | F70-79 | (1) Approximate IQ range of 50 to 69 (in adults, mental age from 9 to under 12 years). Likely to result in some learning difficulties in school. Many adults will be able to work and maintain good social relationships and contribute to society.  (2) Approximate IQ range of 35 to 49 (in adults, mental age from 6 to under 9 years). Likely to result in marked developmental delays in childhood but most can learn to develop some degree of independence in self-car and acquire adequate communication and academic skills. Adults will need varying degrees of support to live and work in the community.  (3) Approximate IQ range of 20 to 34 (in adults, mental age from 3 to under 6 years). Likely to result in continuous need of support.  (4) IQ under 20 (in adults, mental age below 3 years). Results in severe limitation in self-care, continence, communication and mobility.  (5) Other mental retardation.  (6) Unspecified mental retardation. |
| Feeding and eating disorders | F50.8, F98.2 | (1) A feeding and eating disorder of varying manifestations usually specific to infancy and early childhood. It generally involves food refusal and extreme faddiness in the presence of an adequate food supply, a reasonably competent caregiver, and the absence of organic disease. There may or may not be associated rumination.  (2) Psychogenic loss of appetite and other eating disorders with onset during early childhood. |

Supplementary Table 2 Risk of neurodevelopmental disorders in individuals diagnosed with feeding and eating disorder stratified by preterm birth (n=21 637)

|  | | Individuals with FED | |  | Matched controls | |  | | Adjusted  HR (95% CI) |
| --- | --- | --- | --- | --- | --- | --- | --- | --- | --- |
|  | No. Diagnosed | | Incidence rate^*^ |  | No. Diagnosed | Incidence rate^*^ | |  |  |
| **Preterm birth** (n=1241) |  | |  |  |  |  | |  |  |
| Behavioral/emotional disorders | 30 | | 11.91 |  | 38 | 3.88 | |  | 3.25 (1.99-5.33) |
| Attention-deficit/hyperactivity disorder | 13 | | 4.96 |  | 27 | 2.73 | |  | 2.15 (1.09-4.24) |
| Autism spectrum disorder | 16 | | 6.07 |  | 29 | 2.92 | |  | 2.00 (1.06-3.75) |
| Intellectual disability | 10 | | 3.72 |  | 9 | 0.89 | |  | 6.61 (2.23-19.60) |
| **Term birth** (n=20 396) |  | |  |  |  |  | |  |  |
| Behavioral/emotional disorders | 145 | | 8.48 |  | 574 | 2.99 | |  | 2.65 (2.20-3.20) |
| Attention-deficit/hyperactivity disorder | 55 | | 3.09 |  | 342 | 1.77 | |  | 1.61 (1.21-2.16) |
| Autism spectrum disorder | 68 | | 3.83 |  | 232 | 1.20 | |  | 2.99 (2.25-3.95) |
| Intellectual disability | 50 | | 2.76 |  | 68 | 0.35 | |  | 6.53 (4.46-9.58) |

No., number; HR, hazard ratio; CI, confidence interval; ^*^incident rate per 1000 person-years; ^#^ adjusted for parity, parental age, maternal education, maternal origin, maternal cohabitation at birth, parental history of psychiatric disorders before childbirth, and pregnancy complications including diabetes and pre-eclampsia.

Supplementary Table 3 Risk of neurodevelopmental disorders in individuals diagnosed with feeding and eating disorder stratified by maternal psychiatric disorders (n=21 637)

|  | | Individuals with FED | |  | Matched controls | |  | | Adjusted  HR (95% CI) |
| --- | --- | --- | --- | --- | --- | --- | --- | --- | --- |
|  | No. Diagnosed | | Incidence rate^*^ |  | No. Diagnosed | Incidence rate^*^ | |  |  |
| **Maternal psychiatric disorders** (Yes, n=2056) | | | | | | | | | |
| Behavioral/emotional disorders | 38 | | 14.86 |  | 76 | 5.53 | |  | 2.76 (1.85-4.10) |
| Attention-deficit/hyperactivity disorder | 16 | | 5.94 |  | 48 | 3.46 | |  | 1.64 (0.91-2.95) |
| Autism spectrum disorder | 19 | | 7.06 |  | 26 | 1.86 | |  | 4.19 (2.31-7.59) |
| Intellectual disability | 11 | | 3.97 |  | 10 | 0.71 | |  | 4.91 (1.98-12.19) |
| **Maternal psychiatric disorders** (No, n=19 581) | | | | | | | | | |
| Behavioral/emotional disorders | 137 | | 8.03 |  | 536 | 2.85 | |  | 2.79 (2.30-3.39) |
| Attention-deficit/hyperactivity disorder | 52 | | 2.94 |  | 321 | 1.70 | |  | 1.78 (1.32-2.39) |
| Autism spectrum disorder | 65 | | 3.68 |  | 235 | 1.24 | |  | 2.89 (2.17-3.85) |
| Intellectual disability | 49 | | 2.72 |  | 67 | 0.35 | |  | 6.75 (4.59-9.90) |

No., number; HR, hazard ratio; CI, confidence interval; ^*^incident rate per 1000 person-years; ^#^ adjusted for parity, parental age, maternal education, maternal origin, maternal cohabitation at birth, paternal history of psychiatric disorders before childbirth, and pregnancy complications including diabetes and pre-eclampsia.

Supplementary Table 4 Risk of neurodevelopmental disorders in individuals diagnosed with feeding and eating disorders excluding children with low birth weight and lower Apgar score at 5 minutes (<7) (n=20 558)

|  | Individuals with FED | |  | Matched controls | |  | Unadjusted  HR (95% CI) | Adjusted  HR (95% CI) |
| --- | --- | --- | --- | --- | --- | --- | --- | --- |
|  | No. Diagnosed | Incidence rate^*^ |  | No. Diagnosed | Incidence rate^*^ |  |  |  |
| Neurodevelopmental disorders |  |  |  |  |  |  |  |  |
| Behavioral/emotional disorders | 141 | 8.28 |  | 575 | 2.96 |  | 2.82 (2.35 to 3.39) | 2.59 (2.14-3.13) |
| Attention-deficit/hyperactivity disorder | 53 | 3.00 |  | 341 | 1.74 |  | 1.73 (1.29 to 2.30) | 1.56 (1.16-2.09) |
| Autism spectrum disorder | 68 | 3.86 |  | 240 | 1.22 |  | 3.17 (2.42 to 4.14) | 2.97 (2.25-3.93) |
| Intellectual disability | 58 | 2.91 |  | 74 | 0.36 |  | 7.96 (5.64 to 11.22) | 6.65 (4.63-9.56) |

No., number; HR, hazard ratio; CI, confidence interval; ^*^incident rate per 1000 person-years; ^#^ adjusted for parity, parental age, maternal education, maternal origin, maternal cohabitation at birth, parental history of psychiatric disorders before childbirth, and pregnancy complications including diabetes and pre-eclampsia.

Supplementary Table 5 Risk of neurodevelopmental disorders in individuals diagnosed with feeding and eating disorder excluding children diagnosed with cleft lip and cleft palate and other congenital malformations of the digestive system (n=21 348)

|  | Individuals with FED | |  | Matched controls | |  | Unadjusted  HR (95% CI) | Adjusted  HR (95% CI) |
| --- | --- | --- | --- | --- | --- | --- | --- | --- |
|  | No. Diagnosed | Incidence rate^*^ |  | No. Diagnosed | Incidence rate^*^ |  |  |  |
| Neurodevelopmental disorders |  |  |  |  |  |  |  |  |
| Behavioral/emotional disorders | 169 | 8.97 |  | 606 | 3.03 |  | 3.00 (2.53 to 3.55) | 2.81 (2.35-3.35) |
| Attention-deficit/hyperactivity disorder | 66 | 3.37 |  | 366 | 1.82 |  | 1.87 (1.44 to 2.43) | 1.76 (1.34-2.30) |
| Autism spectrum disorder | 76 | 3.88 |  | 259 | 1.28 |  | 3.04 (2.35 to 3.93) | 2.86 (2.19-3.74) |
| Intellectual disability | 58 | 2.91 |  | 74 | 0.36 |  | 7.96 (5.64 to 11.22) | 6.65 (4.63-9.56) |

No., number; HR, hazard ratio; CI, confidence interval; ^*^incident rate per 1000 person-years; ^#^ adjusted for parity, parental age, maternal education, maternal origin, maternal cohabitation at birth, parental history of psychiatric disorders before childbirth, and pregnancy complications including diabetes and pre-eclampsia.

Supplementary Table 6 Risk of neurodevelopmental disorders in individuals diagnosed with feeding and eating disorder compared with age and sex matched controls (n=1 256 989)

|  | | Individuals with FED | |  | Matched controls | |  | Unadjusted  HR (95% CI) | Adjusted  HR (95% CI) |
| --- | --- | --- | --- | --- | --- | --- | --- | --- | --- |
|  | No. Diagnosed | | Incidence rate^*^ |  | No. Diagnosed | Incidence rate^*^ |  |  |  |
| **All combined** |  | |  |  |  |  |  |  |  |
| Emotional/behavioral disorder | 175 | | 8.92 |  | 45 021 | 3.16 |  | 3.00 (2.59-3.49) | 2.85 (2.45-3.32) |
| Attention-deficit/hyperactivity disorder | 68 | | 3.33 |  | 25 955 | 1.81 |  | 1.98 (1.56-2.51) | 1.88 (1.48-2.40) |
| Autism spectrum disorder | 84 | | 4.12 |  | 18 618 | 1.29 |  | 3.33 (2.69-4.13) | 3.04 (2.43-3.80) |
| Intellectual disability | 60 | | 2.89 |  | 5616 | 0.39 |  | 7.98 (6.18-10.29) | 6.39 (4.87-8.38) |
| **Boys** |  | |  |  |  |  |  |  |  |
| Emotional/behavioral disorder | 90 | | 9.80 |  | 30 275 | 4.17 |  | 2.48 (2.02-3.05) | 2.31 (1.87-2.85) |
| Attention-deficit/hyperactivity disorder | 43 | | 4.49 |  | 18 428 | 2.51 |  | 1.89 (1.40-2.56) | 1.80 (1.34-2.43) |
| Autism spectrum disorder | 54 | | 5.63 |  | 14 027 | 1.90 |  | 3.05 (2.33-3.98) | 2.78 (2.11-3.66) |
| Intellectual disability | 32 | | 3.25 |  | 3592 | 0.48 |  | 6.60 (4.66-9.35) | 5.31 (3.72-7.57) |
| **Girls** |  | |  |  |  |  |  |  |  |
| Emotional/behavioral disorder | 85 | | 8.14 |  | 14 746 | 2.11 |  | 4.23 (3.41-5.23) | 3.95 (3.18-4.90) |
| Attention-deficit/hyperactivity disorder | 25 | | 2.31 |  | 7527 | 1.07 |  | 2.41 (1.63-3.57) | 2.22 (1.49-3.32) |
| Autism spectrum disorder | 30 | | 2.78 |  | 4591 | 0.65 |  | 4.64 (3.24-6.64) | 4.33 (3.01-6.24) |
| Intellectual disability | 28 | | 2.56 |  | 2024 | 0.29 |  | 10.43 (7.18-15.15) | 8.86 (6.01-13.05) |

FED, feeding and eating disorders; No., number; HR, hazard ratio; CI, confidence interval; ^*^incident rate per 1000 person-years; ^#^adjusted for parity, parental age, maternal education, maternal origin, maternal cohabitation at birth, maternal smoking status during early pregnancy, parental history of psychiatric disorders before childbirth, and pregnancy complications including diabetes and pre-eclampsia.
